# Metabolic engineering of *Escherichia coli* carrying the hybrid acetone-biosynthesis pathway for efficient acetone biosynthesis from acetate

**DOI:** 10.1186/s12934-019-1054-8

**Published:** 2019-01-14

**Authors:** Hao Yang, Bing Huang, Ningyu Lai, Yang Gu, Zhimin Li, Qin Ye, Hui Wu

**Affiliations:** 1 State Key Laboratory of Bioreactor Engineering, East China University of Science and Technology, 130 Meilong Road, Shanghai, 200237 China; 2 Shanghai Collaborative Innovation Center for Biomanufacturing Technology, 130 Meilong Road, Shanghai, 200237 China; 3 Key Laboratory of Bio-based Material Engineering of China National Light Industry Council, 130 Meilong Road, Shanghai, 200237 China; 4 Key Laboratory of Synthetic Biology, CAS Center for Excellence in Molecular Plant Sciences, Shanghai Institute of Plant Physiology and Ecology, Chinese Academy of Sciences, Shanghai, 200032 China

**Keywords:** Acetate, Acetone, Metabolic engineering, *Escherichia coli*, Gas-stripping, Resting cell

## Abstract

**Background:**

The shortage of food based feedstocks has been one of the stumbling blocks in industrial biomanufacturing. The acetone bioproduction from the traditional acetone–butanol–ethanol fermentation is limited by the non-specificity of products and competitive utilization of food-based substrates. Using genetically modified *Escherichia coli* to produce acetone as sole product from the cost-effective non-food based substrates showed great potential to overcome these problems.

**Results:**

A novel acetone biosynthetic pathway were constructed based on genes from *Clostridium acetobutylicum* (*thlA* encoding for thiolase, *adc* encoding for acetoacetate decarboxylase, *ctfAB* encoding for coenzyme A transferase) and *Escherichia coli* MG1655 (*atoB* encoding acetyl-CoA acetyltransferase, *atoDA* encoding for acetyl-CoA: acetoacetyl-CoA transferase subunit α and β). Among these constructs, one recombinant MG1655 derivative containing the hybrid pathway consisting of *thlA*, *atoDA,* and *adc*, produced the highest level of acetone from acetate. Reducing the gluconeogenesis pathway had little effect on acetone production, while blocking the TCA cycle by knocking out the *icdA* gene enhanced the yield of acetone significantly. As a result, acetone concentration increased up to 113.18 mM in 24 h by the resting cell culture coupling with gas-stripping methods.

**Conclusions:**

An engineered *E. coli* strain with optimized hybrid acetone biosynthetic pathway can utilize acetate as substrate efficiently to synthesize acetone without other non-gas byproducts. It provides a potential method for industrial biomanufacturing of acetone by engineered *E. coli* strains from non-food based substrate.

**Electronic supplementary material:**

The online version of this article (10.1186/s12934-019-1054-8) contains supplementary material, which is available to authorized users.

## Background

Acetone is an important raw material for organic synthesis and a vital solvent in industry. At present, acetone is mainly produced as a co-product in the process of phenol production from cumene, which is a high efficiency and low cost process. However, the petrochemical routes are energy-consuming processes and depend on the unrenewable fossil resources [1]. Bio-based industries has shown advantages over traditional fossil fuel based chemical industry on their environmental impact and resource sustainability. The acetone–butanol–ethanol (ABE) fermentation is a classical acetone production via biological process, although some shortcomings are still existed in this fermentation process.

In the traditional ABE fermentation, the solvent-producing strains (such as *Clostridium* strains) usually use the food-based feedstocks (grain, maize, molasses and so on) as the substrates. Many efforts to improve the ratio and yield of butanol during the ABE fermentation were achieved by using different metabolic engineering strategies [2, 3]. However, a few improvements have been done to increase product specificity. The acetone biosynthetic pathway of *C. acetobutylicum* ATCC 824 was firstly introduced into *E. coli* and the engineered strain accumulated 40 mM acetone in shake-flask culture supplying glucose as carbon source [4]. The CoA-transferase of acetone-synthesis cluster was replaced by one thioesterase which enabled the pathway independent of acetate or butyrate, resulting in 122 mM acetone accumulation in glucose fed-batch culture [5]. A non-oxidative glycolysis pathway was also introduced into *E. coli* by genome expression phosphoketolase from *Bifidobacterium adolescentis* which improved the theoretical acetone yield from 1 to 1.5 mol acetone/mol glucose and obtained 47 mM acetone from glucose in shake-flasks [6]. These works improved the titer and theoretical yield of acetone production from glucose by engineered *E. coli*.

Nevertheless, due to the global food shortage and increase of food price, non-food based substrates, such as crude glycerol, methane, methanol and syngas, were developed as alternative substrates in bio-based industry [7–11]. Acetic acid, a cost-effective non-food based feedstock, can be generated from a variety of cheap sources via chemical or biological ways. A large proportion of acetic acid are produced chemically by liquid-phase methanol carbonylation reaction [12]. Acetic acid also can be synthesized through syngas fermentation by *Clostridium carboxidivorans* [13], photosynthesis from CO_2_ by introducing the self-photosensitization into a nonphotosynthetic *M. thermoacetica* [14], and anaerobic acetogenesis by *M. thermoacetica* [15]. Moreover, acetic acid can also be recovered from lignocellulosic biomass hydrolyzates or pyrolyzates and industrial wastewater [16, 17]. On the other hand, acetate utilization and acetyl-CoA metabolism in *E. coli* have been thoroughly studied, which makes acetic acid more feasible to be used as an alternate carbon. In recent years, acetate has been used to synthesize a series of value-added products, such as medium chain fatty acids [18], lipids [15], ethanol [19], itaconic acid [20], polyhydroxyalkanoates [21], mevalonate [22] and other acetyl-CoA derivatives. The CoA related acetate transportation in the acetone synthetic pathway of *C. acetobutylicum* made it a better substitution than other bio-pathways from acetate to acetone.

In this study, acetone was efficiently synthesized from acetate by constructing acetone synthetic pathway, enhancing acetate assimilation and manipulating central carbon metabolism in engineered *E. coli*. Four different combinations of acetone synthetic pathway were constructed, and their effect on acetone production were tested in *E. coli* MG1655, respectively. With the best acetone producer among them, ACK-PTA (acetate kinase and phosphotransacetylase) enzymes for acetate assimilation were overexpressed, and carbon flux of gluconeogenesis pathway and TCA cycle were reduced to enhance the production of acetone. Finally, resting cells biotransformation together with gas-stripping process was performed to further improve the production and recovery of acetone. The results showed a great potential to replace the fossil fuel based acetone manufacturing with biosynthesis from the renewable feedstock of acetate.

## Results and discussion

### Enhanced acetone biosynthesis from acetate through hybrid synthetic pathway from *C. acetobulylicum* and *E. coli*

**Fig. 1:**
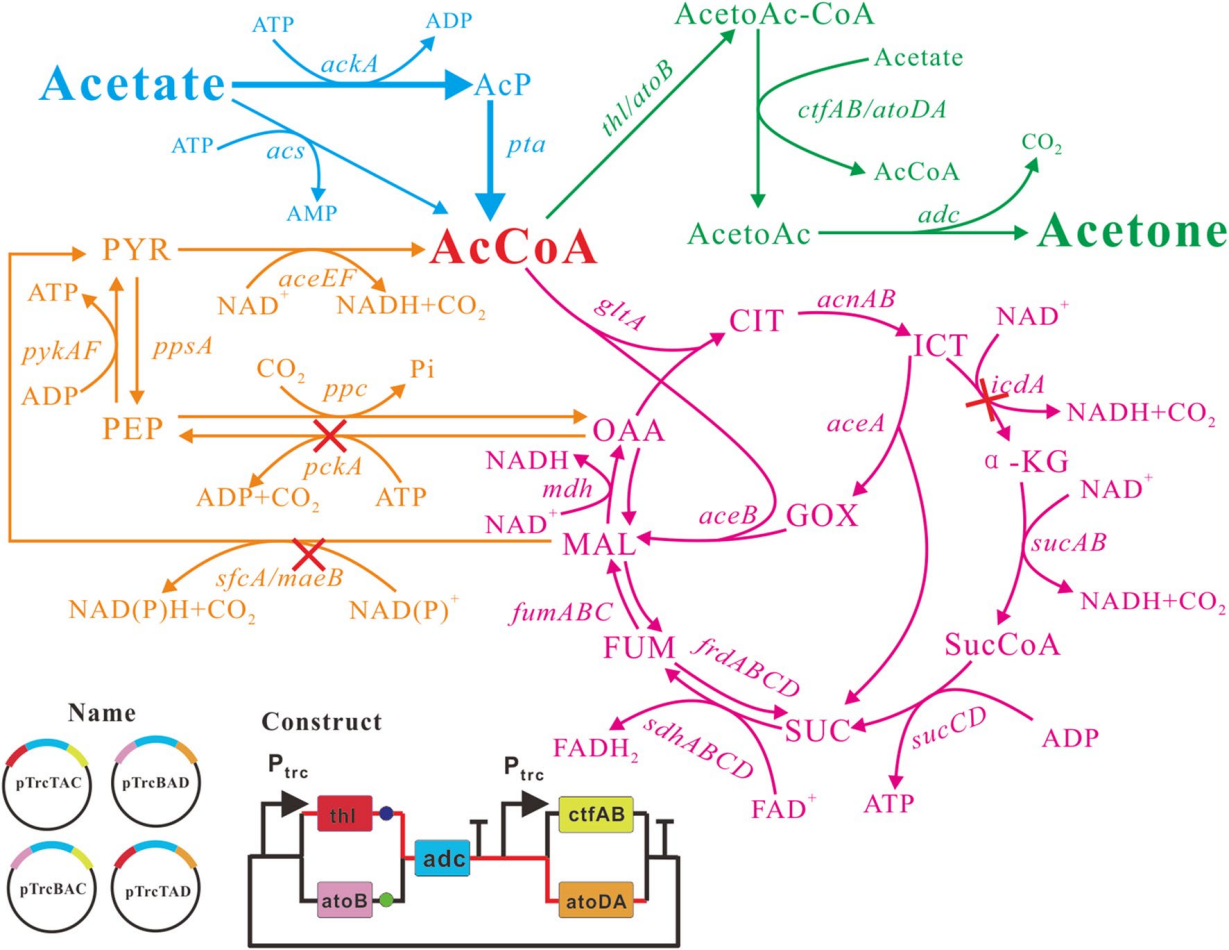
Simplified metabolic pathways of acetone biosynthesis by engineered *E. coli* strain using acetate as carbon source under aerobic condition. Blue arrows represent acetate assimilation pathway, green arrows for acetone synthesis, orange arrows for futile cycle and purple arrows for tricarboxylic acid cycle. Four plasmids containing the acetone biosynthesis pathways were constructed and investigated

**Fig. 2:**
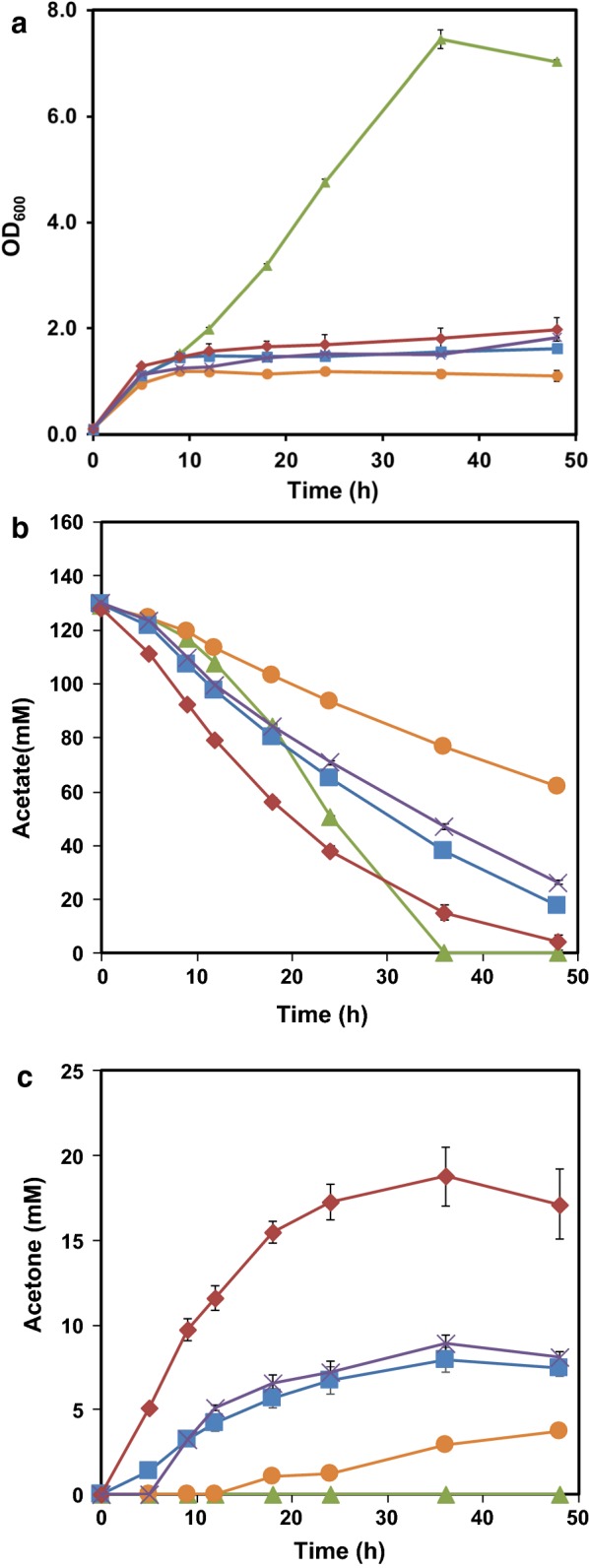
Profiles of cell density (**a**), acetate (**b**) and acetone (**c**) concentrations in cultivation of different strains: MG1655(pTrc99a) (green triangle), MG1655(pTrcBAD) (orange circle), MG1655(pTrcBAC) (**×**), MG1655(pTrcTAC) (blue square), MG1655(pTrcTAD) (red diamond)

In traditional acetone synthetic pathway, acetate is used as a receptor to accept the CoA from acetoacetyl-CoA. However, in *E. coli*, acetate is one of the main by-products from glucose metabolism. Here, we proposed to use acetate directly as the sole carbon source for acetone biosynthesis. In this pathway, acetate is first converted into acetyl-CoA through ACS (acetyl-CoA synthetase) pathway or ACK-PTA pathway. Then, 2 mol of acetyl-CoA are condensed by thiolase to generate 1 mol of acetoacetyl-CoA. Acetoacetyl-CoA transferase transfers the CoA moiety from acetoacetyl-CoA to acetate, and forms acetoacetate and acetyl-CoA, which is part of endogenous acetate utilization pathway. Finally, acetoacetate is catalyzed to form acetone and carbon dioxide by acetoacetate decarboxylase (Fig. 1). Hence, the maximum theoretical yield of acetone is 0.5 mol acetone/mol acetate. Taken from native acetone producing *C. acetobutylicum*, genes *thl*, *adc* and *ctfAB* were first cloned into pTrc99a to generate pTrcTAC. pTrcTAC was then introduced into *E. coli* MG1655 for acetone production. As thiolase (*thl*) and CoA transferase (*ctfAB*) each have endogenous alternatives in *E. coli* (*atoB* for *thl*, and *atoDA* for *ctfAB*), we further constructed three more plasmids (pTrcBAC, pTrcBAD and pTrcTAD) with different combinations of hybrid acetone biosynthesis pathways (Fig. 1). As shown in Fig. 2a, introduction of heterologous acetone biosynthesis pathway in MG1655 impaired the growth of recombinant strains compared to the control strain MG1655(pTrc99a). The cell growth defect suggested that central metabolic pathway did not function well possibly because of lack of enough metabolite precursors. MG1655(pTrc99a) consumed all acetate in 36 h with no acetone production (Fig. 2b, c). Meanwhile, the acetate consumption rate of *atoB* containing strains was relatively low, which indicated Thl was more effective than AtoB for acetate assimilation. MG1655(pTrcTAD) exhibited the highest consumption rate of acetate among the four strains, and the acetone accumulation reached 18.8 mM, which was about 6.5 times, 2.4 times and 2.1 times of that produced by MG1655(pTrcBAD), MG1655(pTrcTAC) and MG1655(pTrcBAC), respectively. In addition, the acetone yield of MG1655(pTrcTAD) was about 1.9 times that of MG1655(pTrcTAC) (0.17 mol/mol vs 0.09 mol/mol) (Fig. 4). The fact that MG1655(pTrcTAD) had a better performance than MG1655(pTrcTAC) for acetone synthesis may be attributed to AtoDA, which has a higher acetate affinity (Km = 53.1 mM) than CtfAB (Km = 1200 mM) [4]. This was consistent with previously reported result of isopropanol production [23]. After acetone biosynthesis pathway from acetate was constructed, the rate of acetate consumption and the acetone yield need to be further improved.

### Effect of enhancing the acetate up-take rate on acetone biosynthesis

**Fig. 3:**
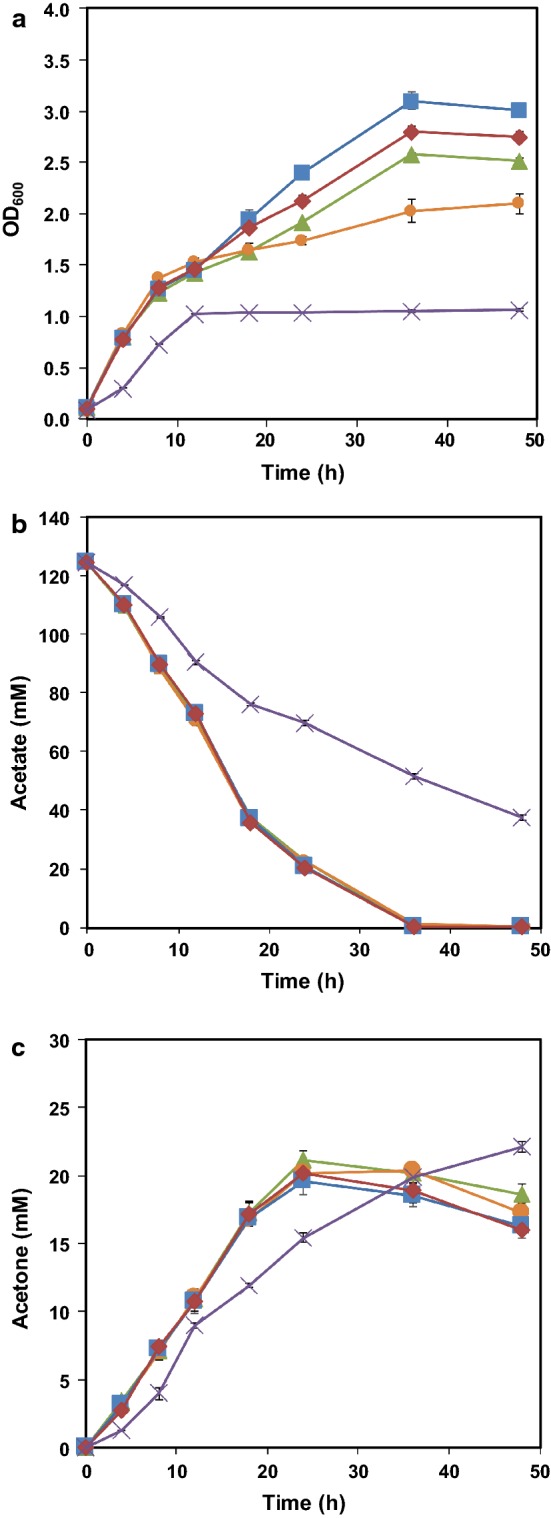
Profiles of cell density (**a**), acetate (**b**) and acetone (**c**) concentrations in cultivation of different strains: HY01(pTrcTAD) (green triangle), HY021(pTrcTAD) (orange circle), HY022(pTrcTAD) (blue square), HY031(pTrcTAD) (red square), HY041(pTrcTAD) (**×**)

**Fig. 4:**
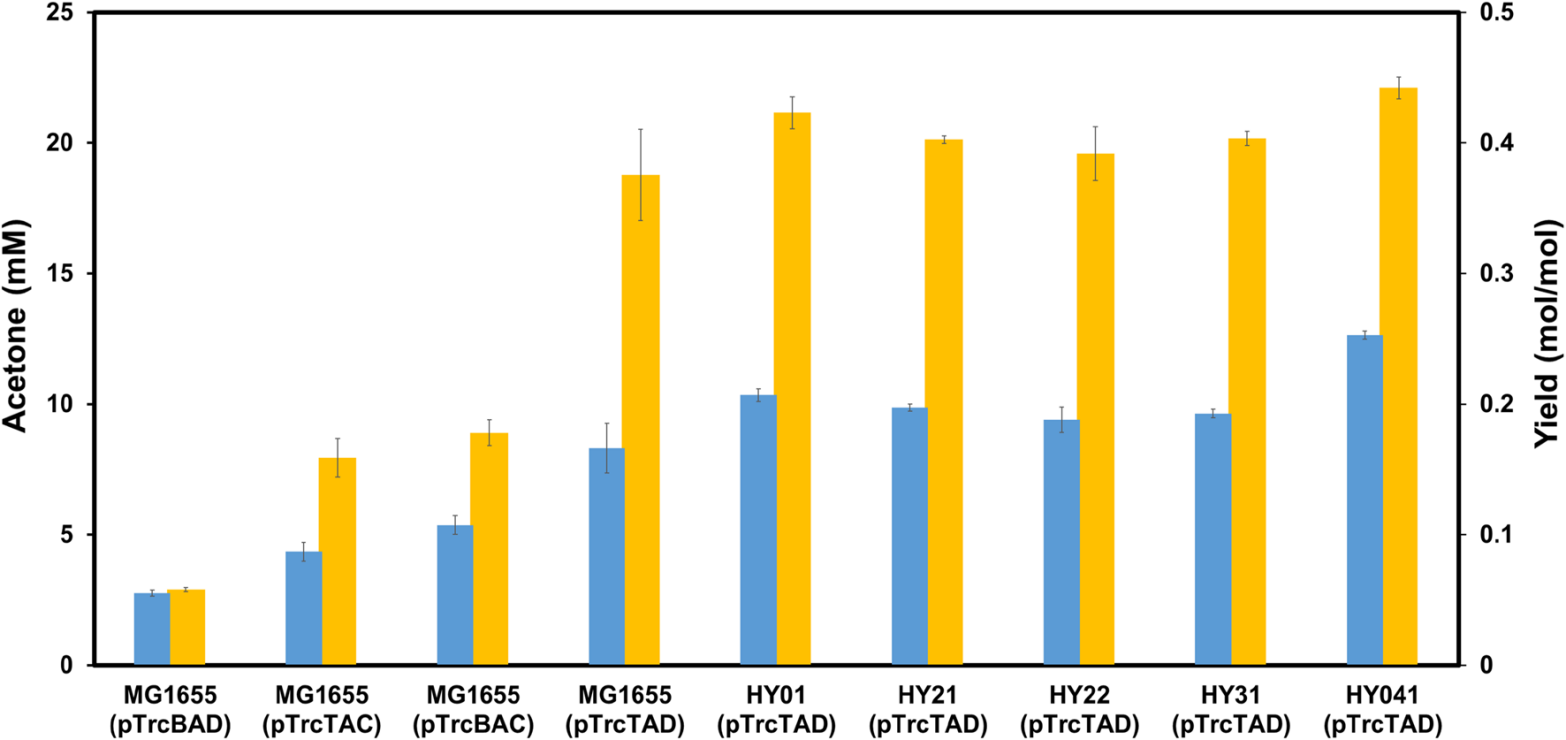
Acetone production and the yield of different metabolically engineered *E. coli* strains in different conditions (yellow square) the titer of acetone; (blue square) the yield of acetone on acetic acid

In *E*. *coli*, the precursor molecule, acetyl-CoA, can be generated not only from pyruvate via several pathways depending on the oxygen conditions, but also from long chains fatty acids and acetate [24]. In order to metabolize different concentrations of acetate to acetyl-CoA, two distinct routes including reversible ACK-PTA pathway (low affinity for acetate) and irreversible ACS pathway (high affinity for acetate) are both existed in *E. coli* [25]. In previous studies, single *acs* gene has been overexpressed to accelerate acetate assimilation [6, 26]. However, for high concentration of acetate, overexpression of *acs* has no significant effect on acetate utilization. In addition, less ATP will be consumed in ACK-PTA pathway due to formation of ADP rather than AMP [24]. Recently, the ACK-PTA pathway was chosen as the target to enhance acetate assimilation during the production of succinate [27] and polyhydroxyalkanoates [19] from acetate. In order to save energy and improve acetone production under high concentration of extracellular acetate, we chose to engineer the ACK-PTA pathway to enhance acetate utilization. In this study, a modified P*trc* promoter (Additional file 1: Table S2) was used to replace the native promoter which was shared by *ack* and *pta* genes in *E. coli* MG1655, yielding strain HY01. The cell growth of the engineered strain was recovered compared to the control one (Fig. 3a). The acetate consumption of HY01(pTrcTAD) in 24 h was about 12.9% higher than that of MG1655(pTrcTAD) (102.3 mM vs 90.6 mM) (Fig. 3b). Meanwhile, the acetone accumulation of HY01(pTrcTAD) reached 1.22 times of that produced by MG1655(pTrcTAD) (21.2 mM vs 17.3 mM) (Fig. 3c). The yield of HY01(pTrcTAD) was increased by 8.4% comparing with that of MG1655(pTrcTAD) (0.21 mol/mol vs 0.19 mol/mol) (Fig. 4). It still can be improved toward the maximum theoretical yield. These results indicated that the enhancement of ACK-PTA pathway was beneficial to acetate utilization and acetone biosynthesis.

### Effect of deletion of PCK and ME on acetone biosynthesis

Blockage of undesired pathway is a common strategy to reduce carbon loss in metabolic engineering. In our previous study, deletion of *pckA* and *maeB* improved succinate production when acetate was used as carbon source [27]. It is also proved that blocking the pathway toward gluconeogenesis can theoretically reduce CO_2_ emission and activate glyoxylate shunt [28]. As is known, phosphoenolpyruvate carboxykinase (*pckA*) is responsible for converting oxaloacetate (OAA) to phosphoenolpyruvate (PEP) which releases CO_2_ and consumes ATP. NADP^+^-dependent malic enzyme (*maeB*) catalyzes malate into pyruvate with CO_2_ forming, too. Meanwhile, these two reactions can also drive carbon flux from TCA cycle to gluconeogenesis pathway. Therefore, *pckA* and *maeB* were deleted in strain HY01, forming HY021 and HY022, respectively. HY031 was further constructed by deletion of *maeB* in HY021. The growth of strain HY021(pTrcTAD) was slightly impaired, while the growth of *maeB* mutant strains were improved significantly. HY022(pTrcTAD) and HY031(pTrcTAD) grew better in acetate than the control strain HY01(pTrcTAD), which were about 26% and 11.3% higher than that of HY01(pTrcTAD), respectively (Fig. 3a). In the meantime, it was interesting that the consumptions of acetate consumption and acetone production among these three engineered strains were similar with the HY01(pTrcTAD) (Fig. 3b), which indicated that the growth yield of the *meaB* mutant strains were higher than that of HY01(pTrcTAD), and less carbon were lost in the form of CO_2_. Moreover, the yield of HY031(pTrcTAD) was slightly higher than HY022(pTrcTAD). Thus, HY031 was chosen to be further modified for better performance.

### Improving the acetone yield by deletion of *icdA*

TCA cycle is one of the center metabolism pathway and plays a significant role in supplement of energy and intermediate metabolites for cell metabolism. The energy generated from TCA cycle is essential for acetate activation. However, in the oxidative arm of TCA cycle, both of the oxidative decarboxylation of isocitrate and α-ketoglutarate will cause carbon loss in the form of CO_2_. In order to reduce the carbon loss, the *icdA* which encodes isocitrate dehydrogenase was deleted, yielding strain HY041. Thus, in HY041, the isocitrate will be metabolized via glyoxylate shunt, which was up-regulated in acetate culture [29]. In this case, 1 mol of NADH can be generated through malate dehydrogenase, which catalyzes malate to oxaloacetate. Meanwhile, under aerobic conditions, 1 mol of NADH can be oxidized to NAD^+^ with generation of about 2 mol of ATP via oxidation respiratory chain [30], which can be used to support cell growth and acetate activation. However, the growth of HY041(pTrcTAD) was inhibited dramatically (Fig. 3a), that may be caused by insufficient supply of α-ketoglutaric acid, a precursor for glutamate biosynthesis [31]. Meanwhile, the rate of acetate consumption was also reduced in HY041(pTrcTAD), and only 43.1 mM was consumed in 48 h (Fig. 3b). However, the titer of acetone was increased to 22.1 mM, which was 19% higher than that of MG1655(pTrcTAD) (Fig. 3c). The yield was improved to 0.25 mol acetone/mol acetate in 48 h, about 1.52 times that of MG1655(pTrcTAD) (Fig. 4). Although the growth of HY041(pTrcTAD) was severely impaired due to *icdA* deletion, it also turned out that the strategy of blocking TCA cycle and redirecting carbon flux to glyoxylate shunt could reduce the carbon loss and increase the yield of acetone.

### Acetone produce by gas stripping coupled with resting cells of engineered *E. coli* strain

**Fig. 5:**
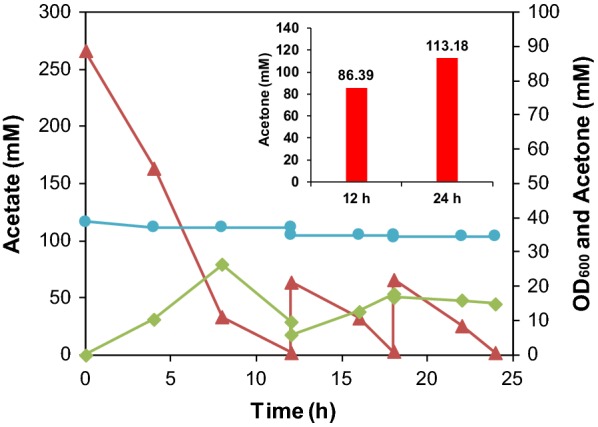
Profiles of cell density (blue circle), acetate (red triangle), and acetone (green triangle) concentrations in the resting cell system of HY041(pTrcTAD) with high cell density (~ 35 OD_600_)

Resting cells has many advantages over growing cell, such as higher cell density, higher product yield and productivity and lower energy requirements, etc. For these reasons, resting cells was applied to investigate the productivity of HY041(pTrcTAD). For cell factory, the toxicity tolerance for substrates and products is of great importance especially for high-density fermentation. The volatility of acetone, which resulted in the decrease of yield in shake flask fermentation, could in turn reduce acetone toxicity taking the advance of in situ product removal technique. To reduce acetone toxicity and avoid leak of acetone, we designed the transformation reactor of resting cells coupled with gas-stripping strategy (Additional file 1: Fig. S1). Since the acetone was produced in the aerobic condition, the air sparging into the broth can serve as the carrying gas of gas tripping. The initial cell density for resting cells biotransformation was around 35 OD_600_ (Fig. 5). In order to obtain high cell density, modified M9 minimal medium containing 5 g/L yeast extract and 10 g/L acetate was used for cell cultivation before resting cells biotransformation. In this stage, 23.0 mM of acetone was produced with a yield of 0.42 mol acetone/mol acetate in 24 h because extra yeast extract was added (Additional file 1: Fig. S2). After 24 h’ cultivation, the cells were harvested for resting cells biotransformation. After 24 h of biotransformation, almost all of the acetate (about 390 mM) was consumed and 113.18 mM of acetone was obtained, with the yield of 0.29 mol acetone/mol acetate (Fig. 5, Additional file 1: Fig. S3). The titer of acetone in the resting cell biotransformation was close to the concentration obtained by using glucose fed-batch cultures, which produced 122 mM acetone in 48 h in *E. coli* [5]. Meanwhile, our results exceeded the titer of acetone achieved in traditional ABE fermentation with *C. acetobutylicum* [32].

## Conclusions

In this study, hybrid biosynthesis pathway using acetate as sole carbon source was constructed by expressing various combinations of genes from *C. acetobutylicum* and *E. coli*. The strain with the combination of *atoDA* from *E. coli* and *thl* and *adc* from *C. acetobulylicum* exhibited the highest titer and yield of acetone. To further improve acetate assimilation and acetone production, a serial of genetic manipulations were conducted to engineer the host *E. coli*. The enhancement of ACK-PTA pathway by exchanging promoter of -*ack*-*pta* improved the acetate assimilation and further increased acetone production significantly. Blocking of gluconeogenesis pathway (deletion of gene *pckA* and gene *maeB*) was proved to have no effect on acetate assimilation but played an important role on cell growth. The redirection of carbon flux into glyoxylate shunt (deletion of gene *icdA*) dramatically improved the yield through bypassing the carbon emission reactions in the oxidative branch of TCA cycle. The engineered *E. coli* strain HY041(pTrcTAD) produced 22.1 mM acetone with the yield of 0.25 mol acetone/mol acetate in 48 h. In the resting cell experiment under high cell density with gas-stripping technology, strain HY041 (pTrcTAD) produced 113.18 mM acetone with the yield increased to 0.29 mol acetone/mol acetate in 24 h. These results suggested great potential of these engineered strains for industrial production of acetone from acetate.

## Methods

### Strains and plasmids

A list of strains and plasmids used are shown in Table 1. Detailed primers for the construction of strains and plasmids are listed in Additional file 1: Table S1. Modified *trc* promotor (P*trc1*, Additional file 1: Table S2) was substituted for the native promotor of *ackA*-*pta* in host strain *E. coli* MG1655 by the one-step PCR-targeting method [33] as same as the following deletions of chromosomal *pckA, maeB* and *icdA*. For the gene deletion in *E. coli*, the DNA fragment containing the kanamycin resistance cassette and homologous arm of recombination was amplified by PCR using the genomic DNA from a single deleted strain (JW3366-1 for deletion of *pckA*, JW2447-5 for deletion of *maeB* and JW1122-2 for deletion of *icdA*) as template.

**Table 1:** Strains and plasmids

Strains and plasmids	Relevant characteristics	Source
*Strains*		
*E. coli* MG1655	Wild type	Laboratory collection
*Clostridium acetobulylicum* ATCC 184	Wild type	ATCC
JW3366-1	BW25113, Δ*pckA:*:Km^R^	Coli genetic stock center (CGSC)
JW2447-5	BW25113, Δ*meaB:*:Km^R^	Coli genetic stock center (CGSC)
JW1122-2	BW25113, Δ*icdA:*:Km^R^	Coli genetic stock center (CGSC)
HY01	MG1655, P*trc*-*ackA*-*pta*	This study
HY021	MG1655, P*trc*-*ackA*-*pta*, Δ*pckA*	This study
HY022	MG1655, P*trc*-*ackA*-*pta*, Δ*maeB*	This study
HY031	MG1655, P*trc*-*ackA*-*pta*, Δ*pckA*, Δ*maeB*	This study
HY041	MG1655, P*trc*-*ackA*-*pta*, Δ*pckA*, Δ*maeB*, Δ*icdA*	This study
*Plasmids*		
pKD4	oriR6Kγ, Km^R^, *rgnB* (Ter)	Datsenko and Wanner [33]
pKD46	*araBp*-*gam*-*bet*-*exo, bla* (Ap^R^), *repA101* (ts), *oriR101*	Datsenko and Wanner [33]
pCP20	Ap^R^, Cm^R^, FLP recombinance	Datsenko and Wanner [33]
pTrc99a	Cloning vector, *trc* promotor, Ap^R^	Laboratory collection
pTrc99a-*trc*-*atoDA*	pTrc99a carries *trc* promoter with *atoD* and *atoA* from *E. coli*	This study
pTrc99a-*trc*-*ctfAB*	pTrc99a carries *trc* promoter with *ctfA* and *ctfB* from *Clostridium acetobulylicum*	This study
pTrcBAD	pTrc99a-*trc*-*atoDA* carries *atoB* from *E. coli* and *adc* from *Clostridium acetobulylicum*	This study
pTrcTAD	pTrc99a-*trc*-*atoDA* carries *thl* and *adc* from *Clostridium acetobulylicum*	This study
pTrcBAC	pTrc99a-*trc*-*ctfAB* carries *atoB* from *E. coli* and *adc* from *Clostridium acetobulylicum*	This study
pTrcTAC	pTrc99a-*trc*-*ctfAB* carries *thl* and *adc* from *Clostridium acetobulylicum*	This study

The construction of recombinant plasmids were described as followed. The genome of *E. coli* MG1655 and *Clostridium acetobulylicum* were used as the PCR templates. Gene segments of *atoB*, *atoDA* from *E. coli* and *thl, adc* from *Clostridium acetobulylicum* were amplified by PCR. The sequence of *lacI* terminator along with the *trc* promoter in the plasmid pTrc99a was amplified as a *trc* promoter cassette by PCR. These resulting DNA fragments were overlapped together to form *trc*-*atoDA*, *trc*-*ctfAB*, *atoB*-RBS-*adc* and *thl*-RBS-*adc* (Additional file 1: Table S3). The optimized RBS between *atoB*-*adc* and *thl*-*adc* were calculated by using online software (https://salislab.net/software/forward). The DNA fragments, *trc*-*atoDA*, *trc*-*ctfAB*, were ligated into linear vector pTrc99a which was digested by *Pst* I and *Hin*d III to form pTrc99a-*trc*-*atoDA* and pTrc99a-*trc*-*ctfAB*, respectively. Then, the DNA fragment of *atoB*-RBS-*adc* was cloned into the plasmid pTrc99a-*trc*-*atoDA* to form pTrc99a-*atoB*-RBS-*adc*-*trc*-*atoDA* (pTrcBAD). The DNA fragment of *thl*-RBS-*adc* was cloned into the plasmid pTrc99a-*trc*-*atoDA* to form pTrc99a-*thl*-RBS-*adc*-Trc-*atoDA* (pTrcTAD). The DNA fragment of *atoB*-RBS-*adc* was cloned into the plasmid pTrc99a-*trc*-*ctfAB* to form pTrc99a-*atoB*-RBS-*adc*-*trc*-*ctfAB* (pTrcBAC). The DNA fragment of *thl*-RBS-*adc* was cloned into the plasmid pTrc99a-*trc*-*ctfAB* to form pTrc99a-*thl*-RBS-*adc*-*trc*-*ctfAB* (pTrcTAC). HB-infusion™ Master mix from Hanbio (Shanghai, China) was used for the above recombination plasmids construction via seamless ligation cloning. PrimeSTAR^®^ HS DNA Polymerase was purchased from Takara (Dalian, China). Restriction enzymes were purchased from NEB (Beijing, China). All resulted plasmids were confirmed by DNA sequencing by Sangon Company (Shanghai, China).

### Media

Luria–Bertani (LB) broth (per liter: tryptone 10 g, yeast extract 5 g, sodium chloride 10 g) was used for the construction of strains and seeds culture. The M9 minimal salts medium consisted of the following components (per liter): 15.12 g Na_2_HPO_4_·12H_2_O, 0.5 g KH_2_PO_4_, 3.0 g, NaCl, 0.5 g MgSO_4_·7H_2_O, 0.011 g CaCl_2_, 1.0 g NH_4_Cl, 0.2 mL 1% (w/v) vitamin B1, and 0.1 mL trace elements solution. The stock solution of trace elements contained the following components (per liter) in 3 M HCl: 80 g FeSO_4_·7H_2_O, 10 g AlCl_3_·6H_2_O, 2.0 g ZnSO_4_·7H_2_O, 1.0 g CuCl_2_·2H_2_O, 2.0 g NaMoO_4_·2H_2_O, 10 g MnSO_4_·H_2_O, 4.0 g CoCl_2_, and 0.5 g H_3_BO_4_. The SMAC media was a modified M9 medium containing 2 g/L of yeast extract and 10 g/L of sodium acetate. Appropriate antibiotics were included at the following concentrations: ampicillin, 100 mg/L; kanamycin, 50 mg/L; chloramphenicol, 34 mg/L.

### Culture conditions

A single colony from a freshly grown plate was inoculated in 3 mL of LB medium at 220 rpm and 37 °C for overnight culture. 1 mL of the primary preculture culture was inoculated (2%, v/v) into 50 mL LB media for aerobic growth in 250-mL conical flask for 10 h. The secondary preculture cultures were inoculated into SMAC media for shake flask fermentation at the ratio of 2% (v/v). Fermentation was carried out at 37 °C, 220 rpm until the OD_600_ reached around 1.0. The expression of the key enzymes of the hybrid acetone biosynthesis pathway were induced by the addition of isopropyl-β-d-thiogalactopyranoside (IPTG) to the final concentrations of 0.1 mM. The incubation temperature was adjusted to 25 °C after adding IPTG. The sterilized H_2_SO_4_ (3 M) was used to adjust the pH of cultures to 7.0 during the fermentation. The volatilization of different concentrations of acetone in flasks were detected (Additional file 1: Fig. S4). All experiments in shake flasks were performed in triplicate.

Resting cells fermentation was performed to increase the productivity of acetone from acetate, using the concentrated resting engineered *E. coli* strain HY041(pTrcTAD). In the resting cell experiments, the preculture conditions were the same as that of the shake-flask fermentation. The modified M9 minimal medium contained 5 g/L yeast extract instead of 2 g/L yeast extract was used for cell cultivation. After cell density (OD_600_) reached about 1.5, the cells were harvested by centrifugation at 6000 rpm and 4 °C for 10 min. The harvested cells were washed twice by the M9 medium without NH_4_Cl. Then, cells pellets were resuspended to 100 mL of sterile NH_4_Cl-free M9 medium containing 20 g/L sodium acetate without adding trace elements and vitamin B1. The resuspended cell broth (35 OD_600_) were transferred into a simplified mini-reactor with automatic pH control system (Additional file 1: Fig. S1). The pH was kept at 7.0 using 3 M H_2_SO_4_, and the temperature was maintained at 25 °C. Due to the air supply of the mini-reactor, the effect of gas-tripping can be achieved during the resting cell experiments. Ten bottles of 100 mL sterilized H_2_O were connected sequentially and linked to the off-gas of the simplified mini-reactor for the acetone collection (Additional file 1: Figure S1). The total amounts of acetone were calculated at 24 h by combining the acetone in all of these bottles with the mini-reactor.

### Analytical methods

Cell density was detected by measuring the optical density of appropriately diluted culture samples at 600 nm (OD_600_). Culture samples were centrifuged for 10 min at 4 °C and 13,000×*g*. The supernatant was then filtered through a 0.22 μm nylon syringe filter. The concentrations of acetone and acetate were detected by HPLC with an aminex HPX-87H ion exclusion column (Bio-Rad, USA), a refractive index detector (RID-10A, Shimadzu Corporation, Kyoto, Japan), a UV detector (SPD-10A, Shimadzu Corporation, Kyoto, Japan), an on-line degasser system (DGU-20A3; Shimadzu) and an LC Solutions system (Shimadzu Corporation, Kyoto, Japan). The mobile phase was 2.5 mM H_2_SO_4_ running at 0.5 mL/min, and the column temperature was operated at 50 °C.

## Additional file

**Additional file 1.** Additional tables and figures.

## Abbreviations

ABE fermentation: acetone–butanol–ethanol fermentation
ACK-PTA: acetate kinase and phosphotransacetylase
ACS: acetyl-CoA synthetase
ADP: adenosine diphosphate
AMP: adenosine monophosphate
ATP: adenosine triphosphate
IPTG: isopropyl-β-d-thiogalactopyranoside
OAA: oxaloacetate
OD: optical density
RBS: ribosome binding site
TCA: tricarboxylic acid cycle
