# Supplementary material for: Metabolic engineering of Escherichia coli carrying the hybrid acetone-biosynthesis pathway for efficient acetone biosynthesis from acetate
Source: Microb Cell Fact. 2019 Jan 14;18:6. doi: 10.1186/s12934-019-1054-8 (PMC6330746; doi:10.1186/s12934-019-1054-8)
Supplement: Supplementary file 1 — Additional file 1. Additional tables and figures. [file 12934_2019_1054_MOESM1_ESM.docx]

Submitted to **Microbial Cell Factories**

**Supplementary Information**

**Metabolic engineering of *Escherichia coli* carrying the hybrid acetone biosynthesis pathway for efficient acetone biosynthesis from acetate**

Hao Yang^1#^, Bing Huang^1#^, Ningyu Lai^1^, Yang Gu^4^, Zhimin Li^1, 2^,Qin Ye^1^, Hui Wu^1,2,3^*

1. State Key Laboratory of Bioreactor Engineering, East China University of Science and Technology, 130 Meilong Road, Shanghai 200237, China

2. Shanghai Collaborative Innovation Center for Biomanufacturing Technology, 130 Meilong Road, Shanghai 200237, China

3. Key Laboratory of Bio-based Material Engineering of China National Light Industry Council, 130 Meilong Road, Shanghai 200237, China

4. Key Laboratory of Synthetic Biology, Institute of Plant Physiology and Ecology, Shanghai Institutes for Biological Sciences, Chinese Academy of Sciences, Shanghai 200032, China.

* Corresponding author: Hui Wu

Telephone: +86-21-64253701

Fax: +86-21-64252250

E-mail: [hwu@ecust.edu.cn](mailto:hwu@ecust.edu.cn)

^#^Both authors contribute equally in this work.

**Contents**

| **Table S1.** Primers for construction of strains and plasmids. | S3 |
| --- | --- |
| **Table S2.** The sequences of original *trc* promoter and the modified *Ptrc*1 promoters | S4 |
| **Table S3.** The sequences of synthetic ribosome binding site with the same translation initiation rate | S5 |
| **Figure S1.** diagram of the micro-bioreactor.  The structure of the micro-reactor: 1, water bath jacket of the micro-reactor; 2, pH electrode; 3, sampling port; 4, valve; 5, gas filter; 6, mini-air generator; 7,water inlet of the water-bath jacket; 8, water outlet of the water-bath jacket; 9, tail gas condensing tube; 10, cold-water inlet of the tail gas condensing tube; 11, cold-water outlet of tail gas condensing tube; 12, 3 M H_2_SO_4_ for pH control; 13, 250 g/L Sodium acetate for supplement of substrate; 14, magnetic stirrer; 15, rotor;16, a set of bottles for collection acetone; 17, bioreactor control system (BG-5, Baoxing Biotech Co., Shanghai, China). | S6 |
| **Figure S2.** Profiles of cell density (A), acetate (B) and acetone (C) concentrations in cultivation of HY041(pTrcTAD) strain. The medium is the modified M9 minimal medium containing 5 g/L yeast extract and 10 g/L acetate. | S7 |
| **Figure S3.** The concentrations of acetone in different collection bottles in 24h. | S8 |
| **Figure S4.** The evaporation of acetone under different concentrations in shake flask fermentation. | S9 |

**Table S1. Primers for construction of strains and plasmids.**

| **Name** | **Primer sequence (5’ to 3’)** |
| --- | --- |
| F-*pckA* | GAATTTCTCCAGATACGTAA |
| R-*pckA* | GCAGGGCACGACAAAAGAAGG |
| F-*maeB* | CAGGCATGGTATTGCTGGAT |
| R-*maeB* | TTCGCTGTGGTGCATAAACT |
| F-*icdA* | atgacggcaaacaatagggt |
| R*-icdA* | gtagaactaccacctgaccg |
| F-P*trc-ack-pta* | AGTGCATGATGTTAATCATAAATGTCGGTGTCATCATGCGCTACGCTCTAGGCCTTTCTGCTGTAGGCTGG |
| R-P*trc-ack-pta* | TTCAGAACCAGTACTAACTTACTCGACATGGAAGTACCTATAATTGATACGGTCTGTTTCCTGTGTGAAAT |
| F-P*trc-ack-pta-*check | AGTGCATGATGTTAATCATAA |
| F-P*trc-ack-pta-*check | TTCAGAACCAGTACTAACTTA |
| F-trc of *atoDA* | tgcactgcaggcgcaacgcaattaatgtgagttag |
| R-trc of *atoDA* | TTGTTTTCATggtctgtttcctgtgtgaaattgtt |
| F-*atoDA* | GAAACAGACCatgaaaacaaaattgatgacattacaagacgccacc |
| R-*atoDA* | CCAAGCTTtcataaatcaccccgttgcgtattcagatc |
| F-trc of *ctfAB* | TCCTCTAGAGTCGACCTGCAGgcgcaacgcaattaatgtgagttag |
| R-trc of *ctfAB* | TAGAGTTCATggtctgtttcctgtgtgaaattgtt |
| F-*ctfAB* | GAAACAGACCATGAACTCTAAAATAATTAGATTTGAAAAT |
| R-*ctfAB* | TCCGCCAAAACAGCCAAGCTTCTAAACAGCCATGGGTCTAA |
| F-*atoB* | AGGAAACAGACCATGGAATTCATGAAAAATTGTGTCATCGTCAGTGCG |
| R-*atoB* | TACTATACTTATCCTTGTTTTTATATGGTCGTTTAATTCAACCGTTCAATCACCATCGC |
| F-*thl* | AGGAAACAGACCATGGAATTCATGAAAGAAGTTGTAATAGCTAGTG |
| R-*thl* | ACCTCCTTATTTCTCGTGTAGTTGCTGCTAGCACTTTTCTAGCAATATTGCTG |
| F-*adc* of *atoB* | AAACAAGGATAAGTATAGTAAGGAGGTTTTCGATGTTAAAGGATGAAGTAATTAAACAA |
| F-*adc* of *thl* | TACACGAGAAATAAGGAGGTAAGGTATGTTAAAGGATGAAGTAATTAAACAAA |
| R-*adc* | CAGGTCGACTCTAGAGGATCCTTACTTAAGATAATCATATATAACTTCAGC |

**Table S2. The sequences of original *trc* promoter and the modified *Ptrc*1 promoters**

| **Promoter** | **Sequence (5’ to 3’)** |
| --- | --- |
| *trc* | TGTTGACAATTAATCATCCGGCTCGTATAATGTGTGGAATTGTGAGCGGATAACAATTTCACACAGGAAACAGACC |
| *Ptrc*1 | TGTTGACAATTAATCATCCGGCTCGTATAATGTGTGGAATTGT**T**A**A**CGG**T**TAACAATTTCACACAGGAAACAGACC |

**Table S3. The sequences of synthetic ribosome binding site with the same translation initiation rate**

| **RBS** | **Sequence (5’ to 3’)** | |
| --- | --- | --- |
| RBS between *thl* and *adc* | CAGCAACTACACGAGAAATAAGGAGGTAAGGT |  |
| RBS between *atoB* and *adc* | ACGACCATATAAAAACAAGGATAAGTATAGTAAGGAGGTTTTCG | |


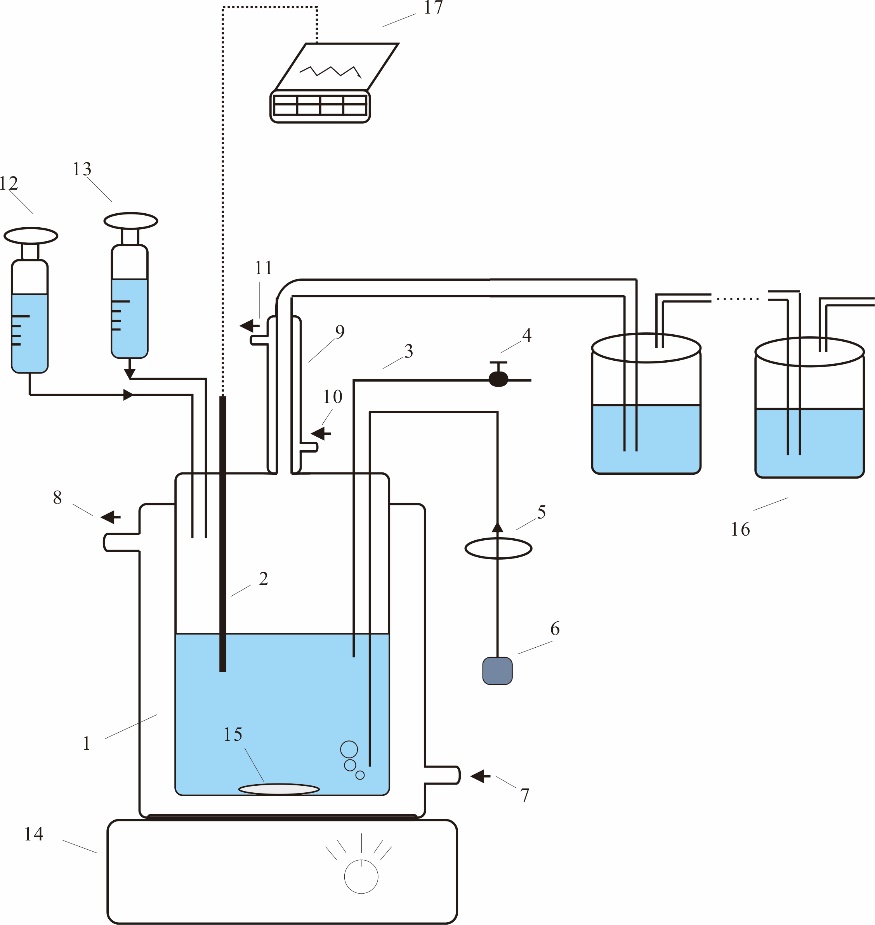


**Figure S1.** diagram of the micro-bioreactor.

The structure of the micro-reactor: 1, water bath jacket of the micro-reactor; 2, pH electrode; 3, sampling port; 4, valve; 5, gas filter; 6, mini-air generator; 7,water inlet of the water-bath jacket; 8, water outlet of the water-bath jacket; 9, tail gas condensing tube; 10, cold-water inlet of the tail gas condensing tube; 11, cold-water outlet of tail gas condensing tube; 12, 3 M H_2_SO_4_ for pH control; 13, 250 g/L Sodium acetate for supplement of substrate; 14, magnetic stirrer; 15, rotor;16, a set of bottles for collection acetone; 17, bioreactor control system (BG-5, Baoxing Biotech Co., Shanghai, China).

**Figure S2.** Profiles of cell density (A), acetate (B) and acetone (C) concentrations in cultivation of HY041(pTrcTAD) strain. The medium is the modified M9 minimal medium containing 5 g/L yeast extract and 10 g/L acetate.

**Figure S3.** The concentrations of acetone in different collection bottles in 24 h.

**Figure S4.**The evaporation of acetone under different concentrations in shake flask fermentation.
